# Changes in axial length in accommodative esotropia patients with minimal hyperopic correction

**DOI:** 10.1371/journal.pone.0203584

**Published:** 2019-01-25

**Authors:** Ye Jin Ahn, Shin Hae Park, Sun Young Shin

**Affiliations:** Department of Ophthalmology and Visual Science, Seoul St. Mary's Hospital, College of Medicine, The Catholic University of Korea, Seoul, Korea; Ohio State University, UNITED STATES

## Abstract

**Purpose:**

To compare the changes of spherical equivalent refractive error (SER) and axial length (AL) for three years in hyperopic children with minimal undercorrection according to the presence of accommodative esotropia (AE).

**Methods:**

A total of 67 hyperopic children were enrolled. The patients were divided into 3 groups and matched by initial age upon examination; esotropic eyes with AE (AE group), fellow eyes with AE (FE group), and right eyes without esotropia (HE group). Changes of SER and AL were serially measured every six months for three years and collected data were compared among the groups.

**Results:**

All three groups underwent significant myopic shift and AL elongation during the follow-up period. However, the least amount of change was found in the AE group. The AE group (-0.96 ± 1.38D) exhibited significantly less change in SER compared to the HE group (-1.76 ± 1.11D) and the FE group (-1.57 ± 1.33D) (both *p*<0.001). Meanwhile, smaller changes of AL were noticeable in the AE group (0.62 ± 0.88mm) compared to the other two groups (HE 0.99 ± 0.29mm; *p*<0.001, FE 0.73 ± 0.65mm; *p* = 0.04). The SER and AL changes were not significantly different between the HE group and FE group.

**Conclusions:**

Esotropic eyes with AE patients with minimal undercorrection exhibited little negative shift of SER and AL elongation compared to not only hyperopic eyes without AE but also fellow eyes with AE.

## Introduction

Co-ordination of the postnatal axial elongation of the eye with the maturation of its refractive components such as corneal power and lens power leads to emmetropization in most human eyes [1]. There has been a focus of interest on refractive errors and the development of ocular components. It is now broadly accepted that the modulation of axial length (AL) in relation to initial refractive error is the most influential factor in emmetropization because corneal power is relatively stable after infancy and plays little role in emmetropization throughout childhood [2, 3]. Crystalline lens power varies inversely with anterior chamber depth to maintain the ratio of the anterior segment to AL. However, the ratio eventually decreases with axial elongation during emmetropization [3–6]. Unlike myopic children who undergo persistent myopic shift with axial elongation, children with hyperopic refractive errors experience less myopic shift and less axial elongation [4].

Accommodative esotropia (AE) is usually treated by prescription of a full correction of hyperopic refractive error in order to obtain satisfactory ocular alignment. However, there are two major conflicts in managing hyperopia–firstly, the spectacle correction of hyperopia may interfere with emmetropization due to visual feedback related to optical defocus [7]. Secondly, refractive correction of hyperopia may improve visual acuity as well as the accuracy of accommodation [8]. Many surveys have tried to explain the reason for failure of emmetropization in children with AE by investigating the effect of hyperopic corrective devices [9–12]. Yang et al. compared the refractive errors of hyperopic children with and without strabismus who received different amounts of spectacle correction [13]. They concluded that full correction of hyperopia may inhibit emmetropization, and that the amount of undercorrection was significantly correlated to change in hyperopic refractive errors.

We manage the conflict between inhibiting emmetropization and improving acuity by undercorrecting our patients by -0.25 to -0.5D based on the cycloplegic refraction with confirming orthotropia at near and distance fixations. However, no studies have been performed regarding the change of AL in AE. Therefore, we aim to compare the changes of spherical equivalent error (SER) and AL between AE children with minimal hyperopic correction and hyperopic children without esotropia.

## Methods

In this observational case series, we retrospectively reviewed the medical records of children with hyperopia from 2012 to 2015. The study protocol followed the guidelines of the Declaration of Helsinki and was approved by the institutional review board of Seoul Saint Mary’s Hospital with waiver of written informed consents. Children who had +2.5 or more diopters hyperopia at the initial visit were included in this study and were followed up at 6 month intervals for at least 3 years. All the children underwent overall ophthalmic examinations at the initial visit including slit-lamp biomicroscopy and fundus photography to exclude any structural abnormalities. Cycloplegic refractions, axial length measurement, and evaluation of ocular alignment status were performed every 6 months. All the data used in this study were collected during the course of routine care. Therefore, we did not obtain written informed consent. AE was defined as an esodeviation that was restored to orthotropia at both near and far fixations through the optical correction of underlying hyperopic refractive errors. Patients with forms of strabismus other than AE (e.g., exodeviation or vertical deviation) or who had a follow-up duration of less than 3 years were not included in the study. Patients with developmental delays, any type of neurologic impairment or other diseases of the visual pathway, or previous extraocular muscle surgery were also excluded from this study. During the follow-up period for patients with AE, spectacles were prescribed on the basis of cycloplegic refraction at the initial visit. When a change in deviation was not observed for at least 1 year, the spectacles were undercorrected by an amount of -0.25 to -0.5D based on cycloplegic refraction at each visit to maintain less than 4PD (prism diopters) esophoria or orthotropia. In patients without AE whose refractive errors were greater than +4D, undercorrected spectacles were prescribed to achieve the best corrected visual acuity.

Refractions were performed using retinoscopy after instillation of 1% cyclopentolate and 0.5% mydriacyl and were reported in terms of SER, calculated as the sphere plus half a cylinder. AL measurements were performed with a Zeiss IOLMaster (Carl Zeiss, Jena, Germany). Multiple measurements were performed along the visual axis of each eye to ensure that test outcomes were repeatable. Ocular alignment was tested by prism alternative cover testing at 4m fixation and 30cm fixation in older children. For preverbal children, the Krimsky or Hirschberg light reflex test was performed. All the tests were performed with and without correction of refractive error. Autokeratometric and biometric measurements were performed on both eyes. However, only the data obtained from the right eye was analyzed, except for comparing changes in SER and AL between esotropic and fellow eyes.

According to ocular alignment, all patients were classified into 3 groups: esotropic eyes with AE (AE group), fellow eyes with AE (FE group), and hyperopic eyes without esotropia (HE; hyperopic eye group). All patients in each group were subdivided according to initial age upon examination and the SER of the more hyperopic eye in a positive number. The patients in each group were then randomly selected from each subgroup to be matched for initial age upon examination and SER of the more hyperopic eye within ±0.5 D. A final total of 67 patients (30 males; mean age 5.48 ± 1.96 years) was included in this study after double matching for age and SER records acquired from the initial 693 patients.

SPSS Statistics 19.0 software (IBM Corporation, Armonk, NY, USA) was used for statistical analysis. The initial and final amounts of SER and AL of each group were compared using one-way analysis of variance. Changes of SER and AL during the follow-up period were compared between the 3 groups using a linear mixed model with Bonferroni’s correction, enabling investigation of the effects of AE on SER and AL through time. A *p* value <0.05 was accepted as statistically significant.

## Results

A total of 67 children were included in the study. The mean AL at the initial visit was 21.22 ± 1.02mm in the AE group, 21.45 ± 1.00mm in the FE group, and 21.66 ± 0.88mm in the HE group, with no significant difference (all *p*>0.05). However, the final SER and AL values were different between HE and AE groups (2.80 ± 1.89D, 3.68 ± 2.01D, p = 0.01; 22.65 ± 0.91mm, 21.84 ± 0.97mm, p = 0.04), while there were no differences in the final SER and AL values between the HE and FE groups or between the AE and FE groups. Data are summarized in Table 1.

**Table 1:** Patient demographics.

	HE group (A) (n = 40)	AE group (B) (n = 27)	FE group (C) (n = 27)	P-value
Gender (male, N)	17	13	13	A vs B (0.76) A vs C (0.76) B vs C (1)
Age (yrs)	5.61 ± 2.02	5.30 ± 1.90	5.30 ± 1.90	A vs B (0.54) A vs C (0.54) B vs C (1)
Undercorrection (D)	-2.11 ± 0.98	-0.31 ± 0.05	-0.27 ± 0.19	A vs B (<0.001) A vs C (<0.001) B vs C (0.68)
Initial SER (D)	4.56 ± 2.14	4.64 ± 1.97	4.48 ± 1.68	A vs B (0.20) A vs C (0.88) B vs C (0.53)
Final SER (D)	2.80 ± 1.89	3.68 ± 2.01	2.91 ± 1.63	A vs B (0.01) A vs C (0.32) B vs C (0.09)
Changes in SER (D)	-1.76 ± 1.11	-0.96 ± 1.38	-1.57 ± 1.33	A vs B (<0.001) A vs C (0.26) B vs C (<0.001)
Initial AL (mm)	21.66 ± 0.88	21.22 ± 1.02	21.45 ± 1.00	A vs B (0.065) A vs C (0.36) B vs C (0.41)
Final AL (mm)	22.65 ± 0.91	21.84 ± 0.97	22.18 ± 1.15	A vs B (0.04) A vs C (0.29) B vs C (0.61)
Changes in AL (mm)	0.99 ± 0.29	0.62 ± 0.88	0.73 ± 0.65	A vs B (<0.001) A vs C (0.16) B vs C (0.04)

N = number; yrs = years; SER = spherical equivalent refractive error; D = diopters; AL = axial length; HE group = hyperopic eyes without esotropia group; AE group = esotropic eyes with accommodative esotropia group; FE group = fellow eyes with accommodative esotropia group

**Fig 1:**
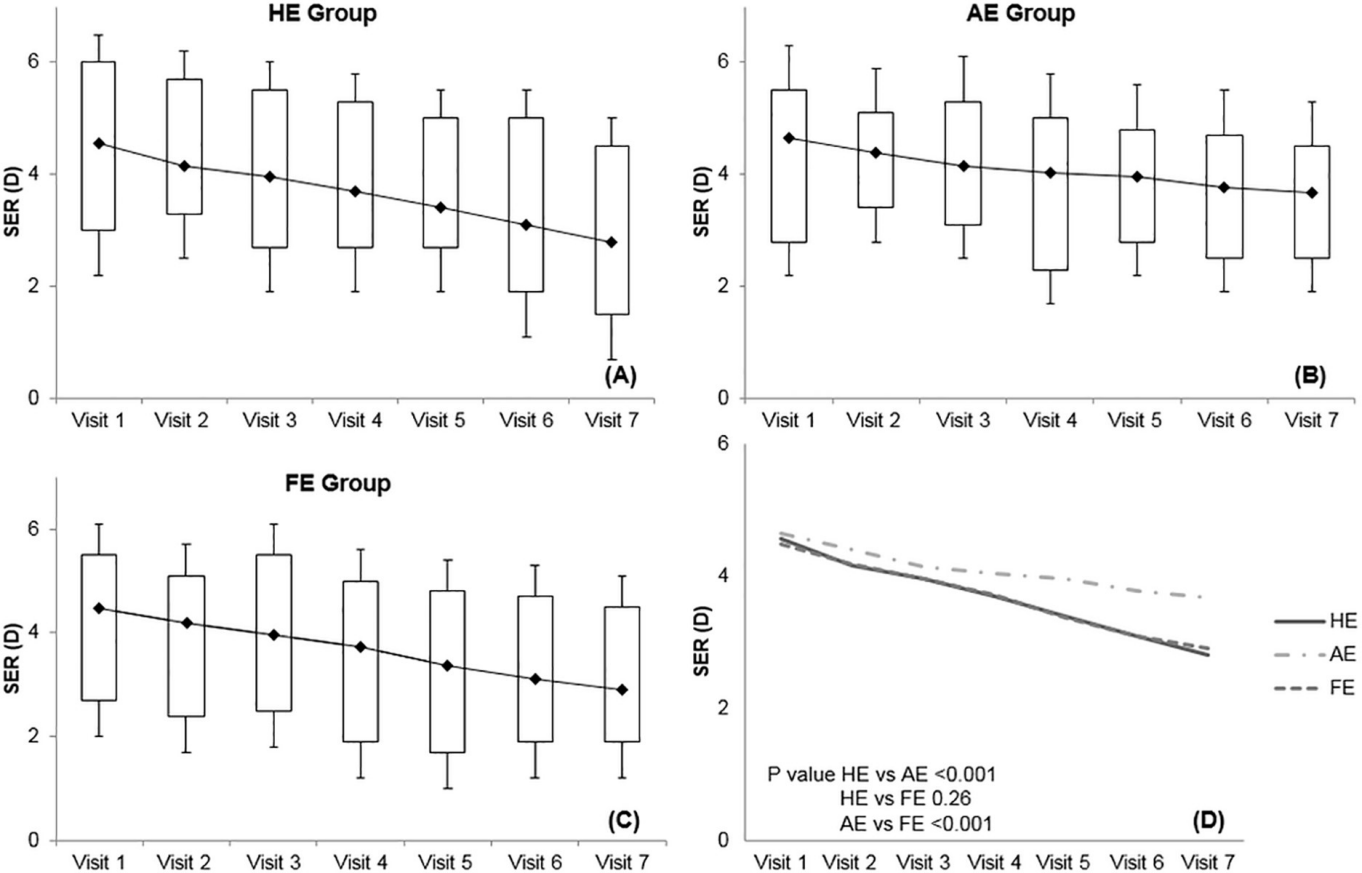
Mean spherical equivalent refractive error change during the follow-up period with 6 month intervals. (A) The no esotropia group (HE group). (B) The esotropic eye group (AE group). (C) The fellow eye group (FE group). (D) Comparison of the 3 groups. Myopic shift was detected in all 3 groups (all *p*<0.05). However, the decreasing tendency of spherical equivalent refractive error over time was smallest in the esotropic eye group, which showed a significant difference with both the no esotropia group and the fellow eye group (*p*<0.001, *p*<0.001, respectively).

Fig 1 shows changes in SER according to time course, which gradually declined during the follow-up period (all *p*<0.05). The AE group exhibited the smallest shift in SER, ending up at +3.68 ± 2.01D at the last follow-up, which was still the most hyperopic SER of the groups. The HE group showed the most rapid decline over time. When compared between groups, the AE group experienced the smallest hyperopic reduction over time compared to the HE (*p*<0.001) and FE groups (*p* = <0.001). There was no difference between the HE and FE groups in SER change over time (*p* = 0.26) (Fig 1D).

**Fig 2:**
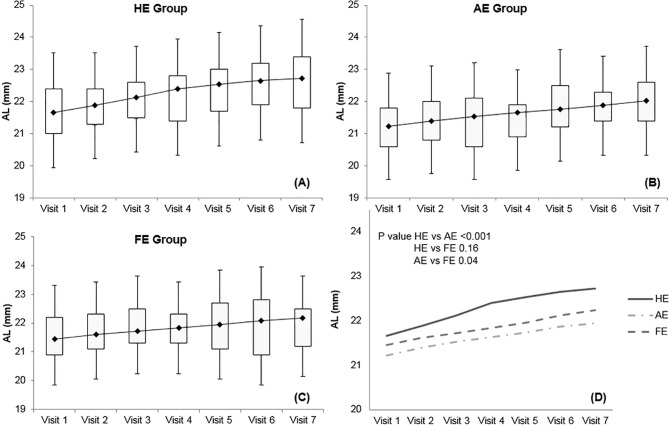
Mean axial length change during the follow-up period with 6 month intervals. (A) The no esotropia group (HE group). (B) The esotropic eye group (AE group). (C) The fellow eye group (FE group). (D) Comparison of the 3 groups. The axial length increased over time in all 3 groups, with meaningful elongation at every visit (all *p*<0.05). However, the esotropic eye group showed the weakest increasing tendency of axial length, and the statistical difference was significant compared to the no esotropia and fellow eye groups (*p*<0.001 and *p* = 0.04, respectively).

AL increased significantly over time in all 3 groups, with substantial elongation at every follow-up (all *p*<0.05) (Fig 2). Patients from the HE group experienced the most dramatic increase in AL, while the most subtle change was found in patients from the AE group. The difference between the two groups was statistically significant (*p*<0.001). The difference was also statistically significant between the FE and AE groups (*p* = 0.04). There was no difference between the HE and FE groups in AL change over time (*p* = 0.16) (Fig 2D).

## Discussion

The esotropic eyes of AE children with minimal undercorrection underwent less negative shift of hyperopic SER and less AL growth compared to fellow eyes with AE and hyperopic eyes without AE.

In the present study, the mean SER gradually decreased through the last follow-up visit in all three groups. This was different from previous studies that reported longitudinal data of SER from children with AE and claimed that there is an initial increase in SER, followed by a myopic shift extending into adulthood [12]. The difference in results may be attributed to the complex interaction of genetic and environmental factors, including the high prevalence of myopia in East Asian children living in big cities with intense nearsighted work and limited outdoor activity [14, 15]. Additionally, the mean age (5.48 ± 1.96 years) of children in our study was relatively higher than previous studies, which included children younger than two years of age.

Previous studies have revealed that full correction of hyperopia may inhibit emmetropization during early and late childhood [12, 16]. Other studies found that spectacle wear itself did not affect refractive changes in AE [10, 17]. Yang et. al[13] compared the change in SER of age-matched children and showed that esotropic children who received full correction of hyperopia achieved less emmetropization than orthotropia children who were fully corrected or undercorrected. Interestingly, the mean negative shift of hyperopia in fully corrected orthotropes was intermediate between that in fully corrected esotropes and undercorrected exotropes/orthotropes. The authors conclude that esotropia and full correction of hyperopia may independently impede axial growth of the eye and the reduction of refractive errors. Likewise, a comparative analysis between fully corrected AE and undercorrected AE was not performed in this study. However, minimally undercorrected eyes with AE exhibited less of a negative shift of SER and elongation of AL than not only hypertropic eyes without AE, but also fellow eyes with AE. These findings suggest that abnormal ocular alignment may affect ocular growth components such as AL, resulting in different final refractive error.

In our study, a negative shift of SER over time was found in all three groups, while the least decreasing tendency was found in the AE group. The difference of SER change was statistically significant between the AE group and the other two groups. Kulp et al.[18] found a negative shift of SER in the amblyopic eye, which remained hyperopic after 10 years of follow-up. Uretmen et al.[19] also derived conforming consequences because the non-dominant eyes with AE were shorter and had more hyperopia than the dominant eyes. This may indicate that anisometropia in some esotropic cases is presumably due to relative emmetropization in the fixating eye, while this process is interrupted in the deviating eye [20]. It has been proposed that strabismus itself may affect axial growth and refractive error development [21]. A greater decrease of SER in the non-amblyopic fellow eye was associated with better ocular alignment, and the greatest decrease was observed in those with orthotropia at baseline, which supports the suggestion that better motor and sensory fusion promotes emmetropization [22, 23]. However, not much is known about the relationship of AE and ocular growth components due to the lack of data on AL change of AE children, which is the strength of the present investigation. In our study, although the AL gradually increased during the total follow-up period in all three groups, the least amount of change in AL was found in the AE group compared to the FE and HE groups. This provides evidence that the actual slowdown in AL causes a less negative shift of SER.

The limitations of our study are as follows. First, this study was retrospective in design. Therefore, the amount of undercorrection was not consistent for different patients. Second, the follow-up period was relatively short. Third, a relatively small number of patients were included in this study. Further prospective studies dealing with a larger sample size and longer follow-up period are required to confirm the results that were suggested in our study.

In conclusion, the results in this study indicate that deviating eyes with AE undergo different ocular growth patterns compared to fellow eyes with AE and hyperopic eyes without AE. The deviating eyes exhibited little negative shift of SER and AL elongation. The effects of initial amount of hyperopia and AL seem to affect future changes in SER and AL, especially in esotropic eyes with AE.
